# Global Landscape of m6A Methylation of Differently Expressed Genes in Muscle Tissue of Liaoyu White Cattle and Simmental Cattle

**DOI:** 10.3389/fcell.2022.840513

**Published:** 2022-03-10

**Authors:** Yunlong Dang, Qiao Dong, Bowei Wu, Shuhua Yang, Jiaming Sun, Gengyuan Cui, Weixiang Xu, Meiling Zhao, Yunxuan Zhang, Peng Li, Lin Li

**Affiliations:** ^1^ College of Animal Science and Veterinary Medicine, Shenyang Agricultural University, Shenyang, China; ^2^ Key Laboratory of Ruminant Infectious Disease Prevention and Control (East), Ministry of Agriculture and Rural Affairs, Beijing, China

**Keywords:** m6A methylation, RNA-seq, muscle growth and development, genetic modification, species

## Abstract

Liaoyu white cattle (LYWC) is a local breed in Liaoning Province, China. It has the advantages of grow quickly, high slaughter ratew, high meat quality and strong anti-stress ability. N^6^ methyladenosine (m6A) is a methylation modification of N^6^ position of RNA adenine, which is an important modification mechanism affecting physiological phenomena. In this study, we used the longissimus dorsi muscle of LYWC and SIMC for m6A-seq and RNA-seq high-throughput sequencing, and identified the key genes involved in muscle growth and m6A modification development by bioinformatics analysis. There were 31532 m6A peaks in the whole genome of LYWC and 47217 m6A peaks in the whole genome of SIMC. Compared with Simmental cattle group, LYWC group had 17,351 differentially expressed genes: 10,697 genes were up-regulated, 6,654 genes were down regulated, 620 differentially expressed genes were significant, while 16,731 differentially expressed genes were not significant. Among the 620 significantly differentially expressed genes, 295 genes were up-regulated and 325 genes were down regulated. In order to explore the relationship between m6A and mRNA expression in the muscles of LYWC and SIMC, the combined analysis of MeRIP-seq and RNA-seq revealed that 316 genes were m6A modified with mRNA expression. To identify differentially methylated genes related to muscle growth, four related genes were selected for quantitative verification in LYWC and SIMC. GO enrichment and KEGG analysis showed that the differentially expressed genes modified by m6A are mainly involved in skeletal muscle contraction, steroid biosynthesis process, redox process, PPAR pathway and fatty acid metabolism, and galactose metabolism. These results provide a theoretical basis for further research on the role of m6A in muscle growth and development.

## Introduction

To date, more than 150 types of posttranscriptional modifications have been identified in the RNA of all living organisms (Boccaletto et al., 2018). The N6-methyladenosine (m6A) modification was discovered in the 1970s and was originally considered to be an abundant nucleotide modification of mRNA in eukaryotic cells (Jia et al., 2013; Yue et al., 2015). Biological functions of m6A modification are mediated by special binding proteins, including methyltransferases, demethylases, and effectors (Zhang et al., 2019). It is involved in various biological processes, such as lipid production and energy metabolism (Zhao et al., 2014; Wang et al., 2015a). In addition, m6A methylation regulates the splicing, expression, decay and translation of mRNA (Wang et al., 2014; Wang et al., 2015b; Xiao et al., 2016). Until recently, little was known about the specific function and mechanism of m6A. Similar to DNA and histone methylation, m6A methylation is also dynamic and reversible in mammals (Wang et al., 2019). It is modulated by several genes, including methyltransferases (METTL3, METTL4 and WTAP) (Liu et al., 2014; Ping et al., 2014), demethylases (ALKBH5 and FTO), (Jia et al., 2011; Zheng et al., 2013) and reading proteins (YTHDF, eIF3 and HNRNPC) (Cao et al., 2016). m6A modification is co installed by a variety of protein complexes (Roundtree et al., 2017). For example, YTHDF2 binds to m6A in mRNA to degrade target genes, while YTHDF1, YTHDF3 and eIF3 promote the translation of m6A containing transcripts (Wang et al., 2014; Wang et al., 2015a; Meyer et al., 2015). As the transferase of writers, METTL3 is composed of catalytic subunit and many other auxiliary subunits. This protein is very important for embryonic growth and development. Embryos lacking METTL3 show pluripotent degradation and damage (Geula et al., 2015). The distribution of mettl3 varies with the type of cell line. In some cases, the change of cell state will lead to the change of its distribution (Knuckles et al., 2017; Xiang et al., 2017). In the cytoplasm, METTL3 itself recognizes the 3′UTR m6A site on mRNA and promotes the formation of translation loop through the interaction with eif3h, so as to promote the protein translation of transcripts (Shi et al., 2019). METTL3 can be functionally regulated by PTM or its protein interaction. It is reported that METTL14 in human cells is phosphorylated at the residue serine399 site on the protein interface with METTL3, indicating that mettl3 has a regulatory function (Wang et al., 2016; Shi et al., 2019). m6A readers protein contains two kinds: one is a stable and direct protein containing YT521-B homology (YTH) domain, and the other is the common RNA binding domain (RBD) (Shi et al., 2019). Both the YTH domain family 1–3 (YTHDF1-3) and the YTH domain containing 1–2 (YTHDC1-2) in humans are stable and directly exercise the reading function of m6A.YTHDF1 and YTHDF3 translation initiation factors promote the translation of target transcripts in cells, and YTHDC2 mediates mRNA stability and translation and regulates cell development (Hsu et al., 2017). The other uses the common RNA binding domain (RBD), such as K homology (KH) domain, RNA recognition motif (RRM) domain and arginine/glycine rich (RGG) domain to preferentially bind the m6A containing region in RNA and exercise the function of m6A reader by regulating the surrounding RNA protein interaction (Shi et al., 2019). Most studies on m6A modification have focused on humans and mice (Dominissini et al., 2012). The m6A methylation is related to obesity (Wang et al., 2020). FTO was the first m6A mRNA demethylase that was discovered. It mediates DNA and RNA demethylation (Jia et al., 2011). The m6A demethylase FTO plays a key role in regulating postpartum growth and energy consumption. A study reported that AMPK regulates lipid accumulation in skeletal muscle cells by regulating FTO expression and FTO-dependent demethylation of m6A (Wu et al., 2017). Research reports on mouse animal models have shown that FTO plays an important role in the regulation of fat mass, adipogenesis, and body weight (Church et al., 2009; Fischer et al., 2009; Church et al., 2010; Gao et al., 2010; McMurray et al., 2013; Merkestein et al., 2015; Ronkainen et al., 2016).

LYWC are excellent beef cattle based on Charolais, breeding the fourth-generation hybrid herd with Liaoning local cattle as the female parent in Liaoning Province, China. A stable population with 93.75% Charolais pedigree and 6.25% local cattle pedigree was formed. LYWC grow quickly, and the slaughter rate was also higher than that of other beef cattle breeds. Due to the large market demand for beef in China, most farms choose LYWC for its excellent production performance. LYWC has wide adaptability, strong stress resistance, and outstanding cold resistance ability and can withstand a low-temperature environment of −30°C. Although LYWC has a better growth rate and slaughter rate, its rough myofiber always influences beef quality directly compared to Simmental and other beef cattle (Jing et al., 2011; Shuangyong et al., 2011). A large number studies have shown that m6A modification plays an important role in regulating lipid production and energy metabolism, inflammatory mechanisms and tumor formation. Based on the necessary functions of m6A modification in regulating gene expression and involving various biological processes, we speculate that m6A modification is involved in beef cattle muscle growth and development. In this study, we aimed to explore the global landscape of differentially expressed m6A methylation genes in muscle tissue between LYWC and SIMC and provide a theoretical basis for further research on the specific regulatory mechanism of unique meat quality and the optimization and selection of LYWC breeds. However, the effect, mechanism, and function of m6A modification on muscle growth and development still needs further research in the future.

## Material and Method

### Sample Collection and Ethics Statement

Three healthy male Liaoyu white cattles and three Simmental cattles were selected for this study and provided the same feed and drinking water during the breeding period. The breeding environment conditions were identical. The average birth weight of LYWC is 40.0 ± 2.0 kg, the average weight at 6 months is 218 ± 5.0 kg, the average weight at 12 months is 366.8 ± 5.0 kg, and the average weight at 24 months is 624.5 ± 5.0 kg. SIMC have an average birth weight of 41 ± 2.0 kg, an average weight of 200 ± 5.0 kg at 6 months, an average weight of 324 ± 5.0 kg at 12 months, and an average weight of 600 ± 5.0 kg at 24 months. They were raised from birth and slaughtered after 24 months.

The longissimus dorsi muscle samples of two breeds of beef cattle were collected after slaughter. A 1 cm^3^ muscle sample was taken from the inner side of the spine near the shoulder area. After that, muscle samples were immediately stored in liquid nitrogen. All experimental procedures were approved and performed according to the guidelines of the Laboratory Animal Management Committee of Shenyang Agricultural University.

### Experimental Procedure

Total RNA was extracted using TRIzol reagent (Invitrogen, CA, United States). The quality and quantity of total RNA were analyzed by Bioanalyzer 2100 and RNA 6000 Nano Labchip kits (Angelon, CA, United States) with value of RIN >7.0. Oligo-dT magnetic beads were used to enrich total RNA with poly(A) mRNA. Approximately 200 µg of total RNA was subjected to isolation of poly(A) mRNA with poly-T oligo-attached magnetic beads (Invitrogen). The lysed RNA fragments were then incubated with m6A-specific antibodies (No. 202003, Synaptic Systems, Germany) in IP buffer (50 mM Tris-HCl, 750 mM NaCl and 0.5% Igepal CA-630) at 4°C for 2 h with BSA (0.5 μg/μl) (1 ml). The mixture was then incubated with protein A beads and eluted with elution buffer (1×IP buffer and 6.7 mM m6A). The eluted RNA was precipitated with 75% ethanol. According to the chain-specific library prepared by the dUTP method, the eluted m6A fragment (IP) and the unprocessed input control fragment were converted into the final cDNA library. The average insert size of the paired-end library was 100 ± 50 bp. We performed paired-end 2 × 150 bp sequencing on the Illumina NovaSeq^™^ 6000 platform of LC-BIO Biotech Ltd. (Hangzhou, China) according to the protocol recommended by the supplier.

### Bioinformatics Analysis Process

First, Cutadapt and local Perl scripts were used to process the data obtained from sequencing to remove low-quality sequences, contaminated sequences, and linker sequences generated by the sequencer to obtain CleanData (Martin, 2011). Fastp (v0.12.6, data quality control doi: 10.1093/bioinformatics/btp616) was used to verify sequence quality, and HISAT2(v2.0.4, alignment reference sequence: doi: 10.1038/nmeth. 3317)) was used to map the read data to the *Bos taurus* genome of cattle with default parameters (Bos taurus_NCBI genome version NA) (USDA ARS) (Kim et al., 2015). The threshold settings of differential peak and differential expression are generally | log2Fc | ≥ 1 and P-val < 0.05. At the same time, qval/fdr is corrected for P-val. Exome Peak (v2.13.2, call peak and diff peak: doi: 10.1093/bioinformatics/btt171) read the IP and input data obtained in the experiment (Meng et al., 2014). This program uses bed or bam format files to identify the m6A peak and visualize it in the UCSC genome browser or with IGV software (http://www.igv.org). MEME and HOMER were used to discover known motifs and locate the peak of the obtained motifs using a Perl script (diff_peak |log2FC|≥1, pval<0.05; call peak log2FC ≥ 1, pval<0.05) (Bailey et al., 2009; Heinz et al., 2010). ChIPseeker analyzes the scanned peak calling and annotates the peak genes (Yu et al., 2015). Then, StringTie (v1.3.4d, assembly quantity: doi: 10.1038/NBT. 3122) was used to perform expression operations on all mRNA in the input database to calculate FPKM (FPKM = [total exon fragments/mapped exon readings (million) × exon length (kB)]) (Pertea et al., 2015). Using the R language package edgeR (v3.20.9, difference analysis: doi: 10.1093/bioinformatics/bt p616), differentially expressed mRNAs with log2 (fold change) > 1 or log2 (fold change)<−1 and *p*-value<0.05 were selected, the reference genome was ARS-UCD1.2 (https://ftp.ncbi.nlm.nih.gov/genomes/all/GCF/002/263/795/GCF_002263795.1_ARS -UCD1.2/) (Robinson et al., 2010). The edger input file was raw counts, and we used edger to analyze the PVAL and qval of the results, calculate fpkm values to measure the expression levels of genes, and compare the fold difference obtained by means of fpkm expression compared to the fold change. Functional enrichment we mapped differential gene functional annotations into different GO term/KEGG pathways by writing our own script, embodying the difference test as a hypergeometric test. The integration of MeRIP-seq and RNA-seq data is related through the annotation of peak, and the qualitative correlation is determined through the up/down of the two parts of regulation. Because exomepeak cannot output the quantification of peak level, it cannot calculate the correlation with the expression.

### Real-Time Fluorescence Quantitative PCR

We tested four different genes with m6A methylation modification for qRT-PCR analysis, they are related to muscle growth and development (Kee and Hardeman, 2008; Otten et al., 2012; Flix et al., 2013; Siddique et al., 2016). We validated the methylation-modified differential genes and used a qRT-PCR kit (Takara, Dalian, China) to reverse-transcribe the total RNA extracted from the muscle into cDNA. SYBR Green (Vazyme-Q711, China) was used to perform real-time fluorescent quantitative PCR according to the instructions. The ACTB gene was used as an internal reference gene to standardize the expression level of genes. Three trials were performed on three LYWC and three SIM muscle samples. Primer 5 was used to design four pairs of primers, the primer list is shown in Table 1. All primers span the end of the gene. The relative expression of differentially expressed genes was calculated by the 2^−△△Ct^ method. The data are expressed as the mean ± standard error (sample number *n* = 3). The *t*-test in SPSS statistical software (version 22.0, Chicago, IL, United States) was used to perform the statistical analyses in the two groups, and the difference was significant when *p* < 0.05.

**TABLE 1:** The primer list.

Gene name	Primer
*MYH6-*F	5′-ACC​CCT​ACG​ACT​ACG​CCT​TC-3′
*MYH6-*R	5′-GTC​AGC​TTG​TAG​ACA​CCG​GC-3′
*MYOM2-*F	5′-CCG​TCC​CTT​CCC​ACC​CTT​AT-3′
*MYOM2-*R	5′-GCT​TGT​CGA​CGT​AGT​AGC​CG-3′
*ACTB-*F	5′-CTC​TTC​CAG​CCT​TCC​TTC​CT-3′
*ACTB-*R	5′-GGG​CAG​TGA​TCT​CTT​TCT​GC-3′
*XIRP1-*F	5′-CAA​ACA​AGA​GGA​ACC​GAC​AGA-3′
*XIRP1-*R	5′-GGC​ATT​GGC​CAT​CCT​TCT-3′
*TNNT1-*F	5′-AGA​AGT​TCC​GGA​AGG​GGG-3′
*TNNT1-*R	5′-ACA​CGC​CAA​GGA​CTC​CCA-3′

## Results and Analysis

### Sequence Statistics and Quality Control

First, the raw data generated by sequencing needed to be preprocessed. Cutadapt filtered out unqualified sequences and removed reads with an adaptor, low quality, and unsure base information. The original sequencing volume, effective sequencing volume, Q20, Q30, and GC content were counted, and appropriate evaluation was conducted. Effective data (Clean Data) was prepared for analysis. In the MeRIP-seq library, we obtained two sets of muscle sample data reads. Three biological replicates were performed in each group, and the effective reading data were as follows: LYWC group: 75018648, 63985000 and 73353244; SIM group: 34991588, 42841030 and 50385720. The percentages of valid data (Clean Data) in the two groups of data were 71.85, 71.45, and 73.96% in the LYWC group and 90.24, 91.73, and 96.68% in the SIM group, respectively. In the RNA-seq library, we obtained two sets of muscle sample data reads, each of which was subjected to three biological replicates: the effective read data were as follows: LYWC group: 53535846, 73285174, and 42315990; SIM group: 70936986, 96166358 and 66577584. The percentages of valid data in the two groups of data were 96.52, 96.42, and 96.24% in the LYWC group and 89.09, 98.52, and 90.23% in the SIM group, respectively (Table 2). In Table 2, the proportion of Q20% bases with a quality value ≥20 (sequencing error rate is less than 0.01) and the proportion of Q30% bases with a quality value ≥30 (the sequencing error rate is less than 0.001) are shown.

**TABLE 2:** Summary of reads quality control.

Sample_	Raw_Reads	Valid_Reads	Valid%	Q20%	Q30%	GC%
LYWC1_IP	75018648	69503364	71.85	98.71	96.25	58.98
LYWC2_IP	63985000	58996716	71.45	98.64	96.03	59.02
LYWC3_IP	73353244	69624344	73.96	98.81	96.37	57.88
SIM1_IP	34991588	32396532	90.24	98.30	95.29	57.62
SIM2_IP	42841030	40364782	91.73	97.87	94.15	58.51
SIM3_IP	50385720	49785152	96.68	98.20	94.82	57.03
LYWC1_input	53535846	52583702	96.52	97.43	92.96	57.89
LYWC2_input	73285174	71862022	96.42	97.35	92.81	57.35
LYWC3_input	42315990	41541788	96.24	97.40	92.95	56.52
SIM1_input	70936986	68695324	89.09	98.18	94.97	58.41
SIM2_ input	96166358	95102418	98.52	98.11	94.57	56.15
SIM3_ input	66577584	64093630	90.23	97.98	94.45	57.91

### Map Data to Genome

**FIGURE 1:**
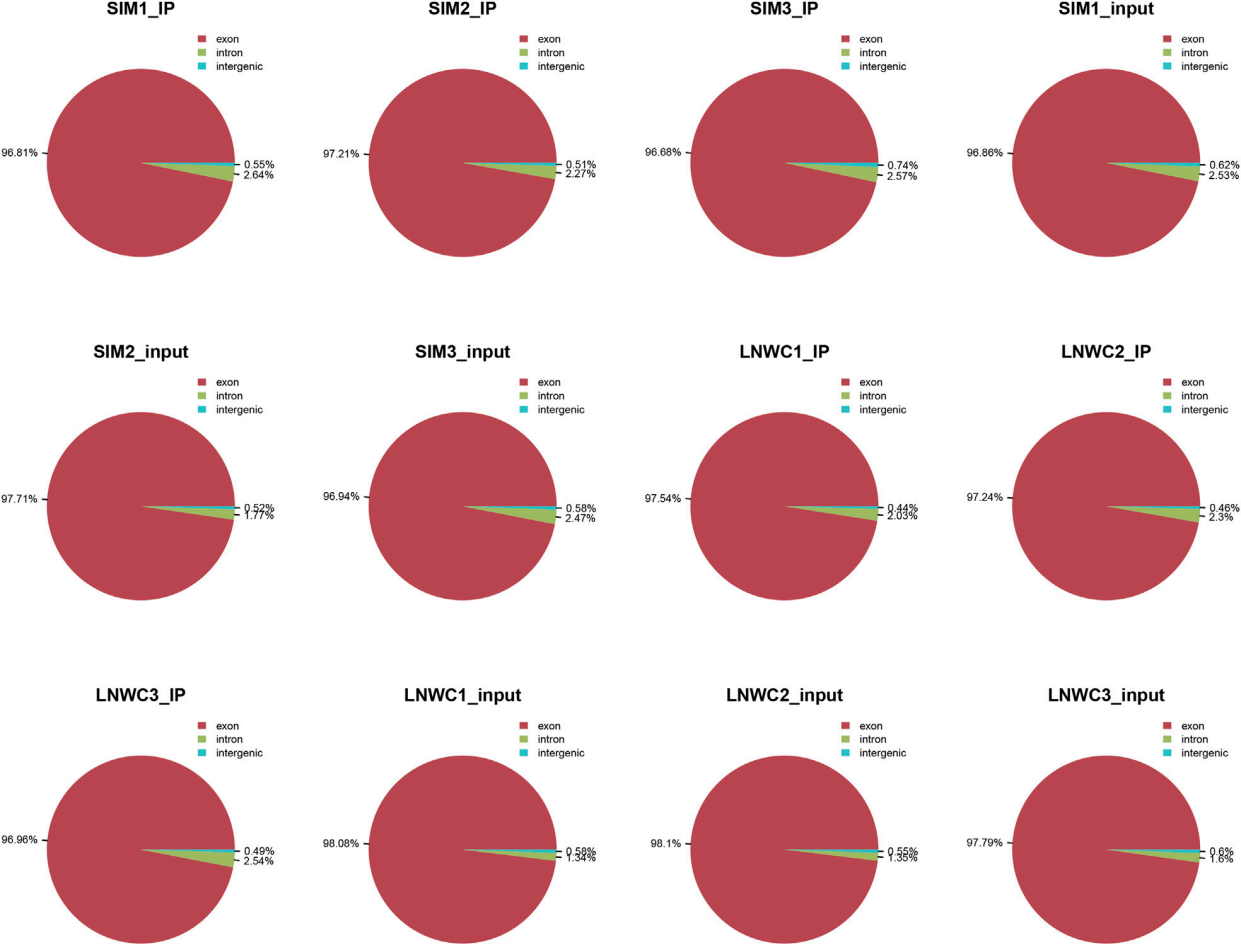
Comparison of the regional distribution with reference to the genome.

We used HISAT2 for reference genome comparison of the preprocessed valid data and mapped reads to the *Bos taurus* cattle (Bos taurus_NCBI, version NA) genome with default parameters. By comparing the obtained reads with the reference genome sequence, we can perform detailed statistics on the data obtained by sequencing and its distribution in the genome. In the m6A-seq library, the IP samples of the longissimus dorsi muscle of beef cattle are LYWC_IP and SIM_IP, and we performed three replicates for each set of samples. The LYWC_IP effective data mapping read rates were 94.13, 94.87, and 95.20%; the SIM_IP effective data mapping read rates were 92.92, 90.97, and 92.30%. In the RNA-seq library, the longissimus dorsi samples of beef cattle are LYWC_input and SIM_input, and we performed three replicates for each set of samples. The effective data mapping read rates of LYWC_input are 96.95, 96.89, and 96.84%, respectively; the effective data mapping read rates of SIM_input are 94.95, 97.79, and 93.68%, respectively. Unique mapped reads are shown in Table 3. According to the regional distribution information of the reference genome, it can be defined as alignment to three parts of exon (exon), intron (intron) and intergenic (intergenic region).In general, the percentage of the sequence alignment to the exon region should be the highest. The results of this experiment showed that the IP samples of Liaoyu white cattle accounted for 97.54, 97.24 and 96.96% in the exon region; the ratios of the input samples were 98.08, 98.10 and 97.79%, respectively. The IP samples of Simmental cattle accounted for 96.81, 97.21 and 96.68% in the exon region; the ratios of the input samples were 96.86, 97.71 and 96.94%, and the results are shown in Figure 1.

**TABLE 3:** Summary of reads mapped to the cattle reference genome.

Sample	Valid reads	Mapped reads	Unique mapped reads	Multi mapped reads
LYWC1_IP	69503364	65423613 (94.13%)	53565538 (77.07%)	11858075 (17.06%)
LYWC2_IP	58996716	55968831 (94.87%)	46290788 (78.46%)	9678043 (16.40%)
LYWC3_IP	69624344	66284434 (95.20%)	53585255 (76.96%)	12699179 (18.24%)
LYWC1_input	52583702	50979857 (96.95%)	32727624 (62.24%)	18252233 (34.71%)
LYWC2_input	71862022	69628680 (96.89%)	44600306 (62.06%)	25028374 (34.83%)
LYWC3_input	41541788	40229546 (96.84%)	26453120 (63.68%)	13776426 (33.16%)
SIM1_IP	32396532	30103081 (92.92%)	24045688 (74.22%)	6057393 (18.70%)
SIM2_IP	40364782	36721102 (90.97%)	29120560 (72.14%)	7600542 (18.83%)
SIM3_IP	49785152	45949312 (92.30%)	34638134 (69.58%)	11311178 (22.72%)
SIM1_input	68695324	65225662 (94.95%)	53655224 (78.11%)	11570438 (16.84%)
SIM2_input	95102418	93001053 (97.79%)	63683053 (66.96%)	29318000 (30.83%)
SIM3_input	64093630	60045990 (93.68%)	47919015 (74.76%)	12126975 (18.92%)

### Identification of m6A Modification Sites and Analysis of Differentially Methylated Genes

**FIGURE 2:**
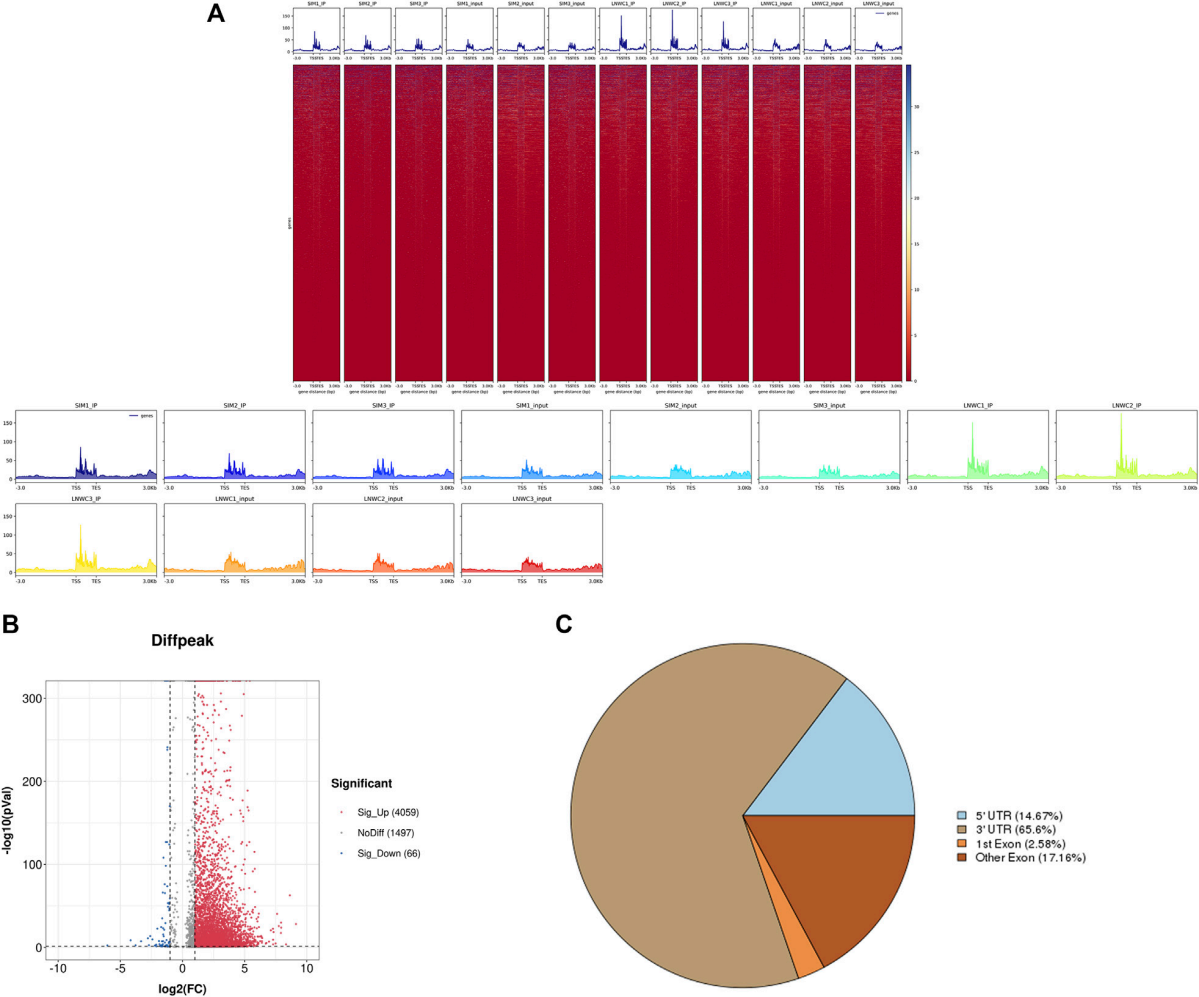
**(A)** Heat map of the enrichment of reads near the TSS and the peak distribution near the TSS at the start site of gene transcription. **(B)** The distribution of peaks of original differences in gene function. **(C)** Distribution of differential peak on gene functional elements.

Use peak-calling software, the R language toolkit exomePeak, was used to scan the m6A peak in the entire genetic dataset. Based on the identification of the IP and input libraries, biological information such as the position and length of the peak in the gene can be obtained. The call peak portion we choose P-val < 0.05, and the diff peak and diff expression portions generally choose|log2 fc| ≥ 1 and P-val < 0.05. We counted and combined all the samples and the degree of enrichment of the reads near the gene transcription start site (TSS). The peaks that could be combined near the TSS are represented in the form of a heat map, as shown in Figure 2A. ChIP seeker software was used to annotate the different peaks and perform GO and KEGG enrichment analyses. In general, the default *p* < 0.05 was the filter condition of the peak. Compared with Simmental cattle group, we screened 5631 difference peaks in Liaoyu white cattle group, of which 4,059 m6A peaks were significantly up-regulated and 66 m6A peaks were significantly down regulated, as shown in Figure 2B. The distribution of m6A peak in transcripts was analyzed. Analysis of the distribution of m6Apeak in the transcript was performed. We divided the transcript into four parts: 5′-UTR, 3′-UTR, first exon and other exons. It was used to analyze the distribution of different peaks in the original gene function, as shown in Figure 2C. As shown in Table 4, the m6A modification was mainly enriched in the 3′-UTR, and we report the top 20 differences m6Apeak. Difference factor <1 indicates hypomethylation, and difference factor >1 indicates hypermethylation.

**TABLE 4:** The top 20 differentially expressed m6A peaks.

Gene name	Fold change	Regulation	Chromosome	Peak region	Peak start	Peak end	p-value
GLUL	560.28	Up	16	5′ UTR	63478467	63478646	9.99E−288
LOC112445778	398.93	Up	Un	Exon	987	1142	1.6E−82
BRICD5	321.80	Up	25	3′ UTR	1740015	1740254	1E−221
WASF2	245.57	Up	2	Exon	125880669	125880759	0.000000000025
SNRPA	243.88	Up	18	3′ UTR	50032385	50032564	0.000000000000000001
PTGDS	242.19	Up	11	3′ UTR	106024413	106024969	1.6E−87
COPZ1	215.27	Up	5	5′ UTR	25742023	25742113	3.2E−32
JSP.1	195.36	Up	23	Exon	28666476	28666821	1E−24
PPP1R3B	179.77	Up	27	5′ UTR	25190141	25190261	0.00059
PAFAH1B1	160.90	Up	19	5′ UTR	23512920	23513159	6.3E−27
TYW5	0.02	Down	2	3′ UTR	88497265	88497444	0.000026
TACC1	0.06	Down	27	Exon	33995126	33998354	0.00000046
MTSS1	0.07	Down	14	Exon	15550578	15550728	0.00000023
MAML1	0.10	Down	7	Exon	1553848	1553937	0.000087
ARHGAP21	0.15	Down	13	Exon	25465042	25465341	0.0000000000000032
RTF1	0.15	Down	18	Exon	36887325	36887415	0.0000074
ATXN1L	0.17	Down	18	Exon	39240553	39240912	0.0000000000000000005
ANKRD11	0.19	Down	18	Exon	14335439	14335618	0.000000013
EVI5L	0.22	Down	7	5′ UTR	16608242	16608302	0.000000000002
MASP1	0.22	Down	1	3′ UTR	80048359	80048508	0.0000000087

### Motif Analysis

**FIGURE 3:**
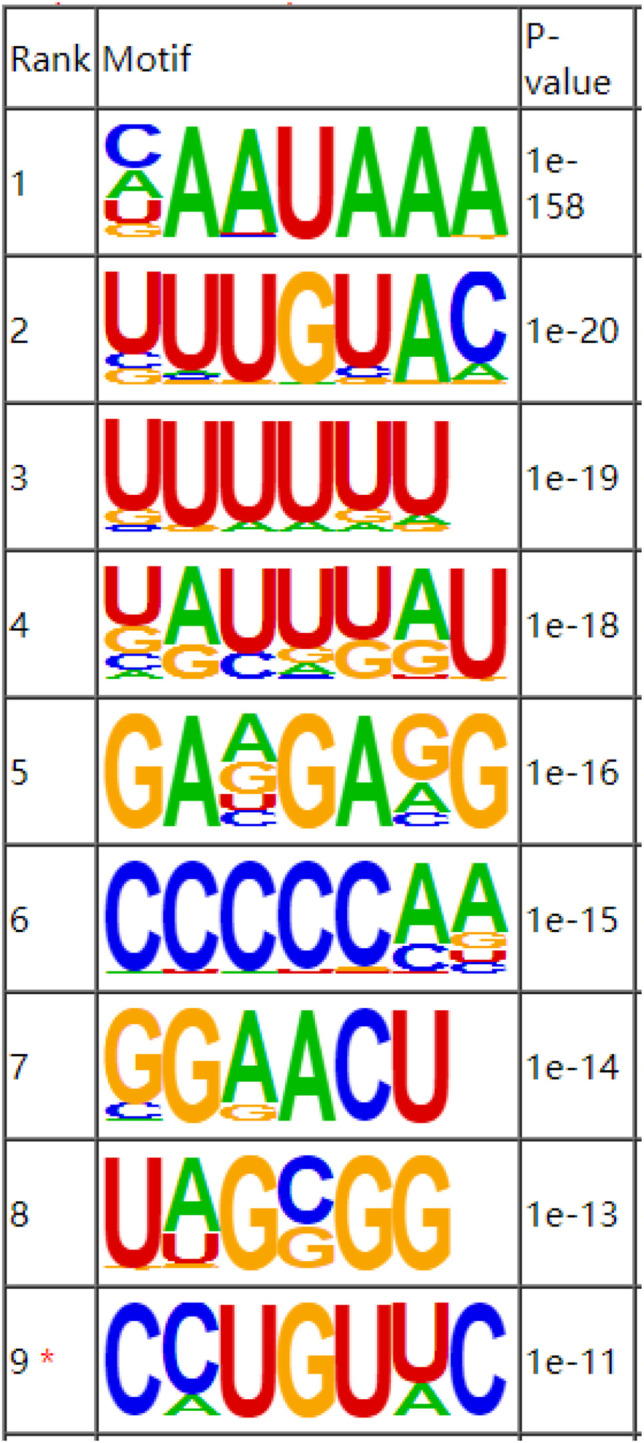
Sequence showing the motifs with significant differences in muscle samples at the m6A peak.

As a dynamic modification phenomenon, RNA methylation is mainly accomplished by the combined action of multiple methylases and methylation binding site motifs. A motif is a nucleotide sequence pattern with biological significance, and the sequence has a high degree of conservation. The methylases involved in the process of RNA methylation recognize the motifs in the gene to generate methylation and regulate gene expression. The motif software MEME was used to search for more credible motifs in the peak area and obtain information about the width, E-value, and location of each motif. We performed motif prediction on each set of samples, and the results are shown in Figure 3. A motif structure that is reported commonly in RNA modifications are RRACH (where R = A or G, H = A, C or U).

### Whole Gene Analysis and Differential Gene Analysis

**FIGURE 4:**
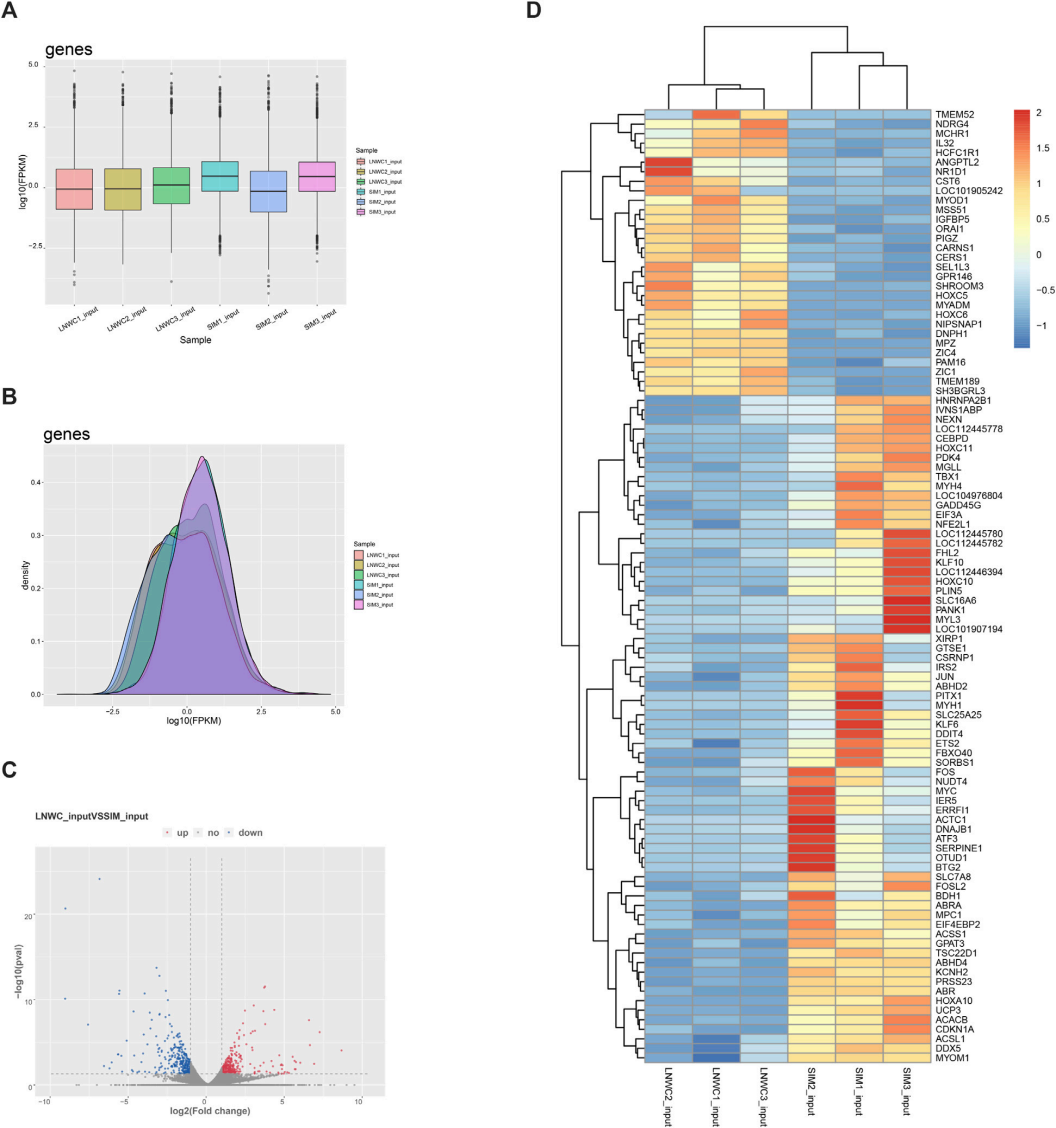
**(A)** Gene expression cassette diagram. **(B)** Gene expression density diagram. **(C)** Differential gene expression volcano diagram. In the figure, log2 of the fold change is the horizontal coordinate, and −log10 (*p*-value) is used as the vertical coordinate. The horizontal coordinate represents the gene expression in different samples; the vertical coordinate represents the significant difference in gene expression. Among them, red represents upregulated significantly differentially expressed genes, blue represents downregulated significantly differentially expressed genes, and gray represents nonsignificantly differentially expressed genes. **(D)** LYWC and SIMC gene heat map. Using zscore standardization, the expression levels of genes in different samples can be compared horizontally. From blue to red, the expression amount of genes ranges from low to high.

The expression level of genes is mainly measured by RPKM (reads per kilobase of exon model per million mapped reads) or FPKM (fragments per kilobase of exon model per million mapped reads) to measure the abundance value of gene expression. In our research, we chose FPKM to report the expression abundance values of different samples of known genes. Compared with Simmental cattle, Liao yu white cattle detected 17,351 differentially expressed genes, 620 genes were significantly different and 16,731 genes were not significantly different (|log2fc| ≥ 1 and *p* < 0.05). Among the differentially expressed genes, 10,697 genes were upregulated and 6,654 genes were downregulated. Among the 620 significantly differentially expressed genes, 295 were up-regulated and 325 were down regulated. Table 5 shows the top 20 differentially expressed genes we screened. Among the top 20 differentially expressed genes, there are 10 up-regulated genes and 10 down-regulated genes. We used Figures 4A,B to show the gene expression and expression density. We plotted the overall distribution statistics of differentially expressed genes, as shown in Figure 4C. Figure 4D shows the gene heat map between LYWC and SIM samples.

**TABLE 5:** The top 20 differentially expressed genes.

Gene name	Fold change	Regulation	Locus	Strand	*p*-value
ZIC4	408.70	Up	Chr1:120900467-120920588	+	0.0000893270205096843
ZIC1	154.75	Up	Chr1:120896651-120900435	−	6.48985151731775E−07
KCNG2	121.11	Up	Chr24:614160-636366	−	0.00333415305292261
HOXC5	100.57	Up	Chr5:25998379-25999826	−	0.0000215125233082445
LOC101905242	96.40	Up	Chr1:42016839-42017535	+	2.6240518996526E−08
LOC104972118	65.46	Up	Chr4:70745406-70752937	−	0.00252204598175991
HOXC4	53.97	Up	Chr5:25977635-25994974	−	0.00209569391674476
EMX2	53.11	Up	Chr26:37830529-37837034	+	0.0144795011020604
ABI2	51.26	Up	Chr2:91544895-91646688	+	0.0142005975927657
COL23A1	50.83	Up	Chr7:39317415-39719111	−	0.00151425556715255
LOC112445780	0.0019054052765261	Down	Chrun:1379-3188	−	8.00798384058217E−11
PITX1	0.00192374812736059	Down	Chr7:46474414-46480622	−	2.15915187958383E−21
LOC112445782	0.00526541615536884	Down	Chrun:37307-41037	−	8.49182655839351E−08
HOXC10	0.00878243398141918	Down	Chr5:26042721-26047450	−	7.56247125923241E−25
COL22A1	0.0106909776847475	Down	Chr14:4095051-4319199	+	0.00608209941205491
LOC101905017	0.013655843197307	Down	Chr11:100029843-100030458	−	0.0111876548271218
COMP	0.0150500815850192	Down	Chr7:4422721-4430541	+	0.00230163465282101
BOLA	0.0199056647166198	Down	Chr23:27943375-27950488	+	0.000265837339817353
LYL1	0.0201706382454435	Down	Chr7:12473687-12479882	+	0.000260783474606451
OTUD1	0.0208958121373525	Down	Chr13:24387754-24390638	+	2.18756401110904E−11

### Joint Analysis of Differentially Expressed Genes and Differentially Expressed Genes

**FIGURE 5:**
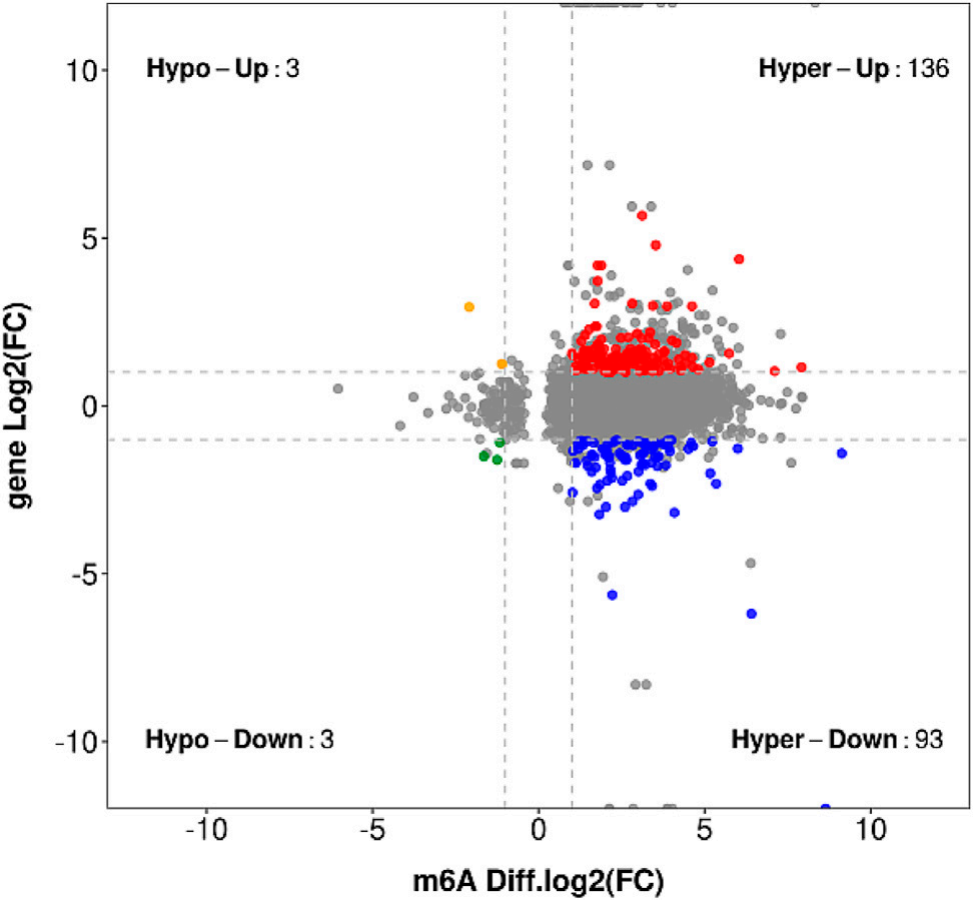
The result obtained by taking the intersection of the gene where the significant difference m6A peak is located and the significant difference expression gene is used, and a stricter screening threshold is used.

In the entire transcriptome sequencing, we found that there were upregulated and downregulated genes. In the MeRIP-seq sequencing results, according to the changes in abundance, we found that the methylation of the gene itself was upregulated and downregulated. Therefore, we combined the correlation analysis of the two sequencing results to compare and analyze the transcription level and methylation level. In the samples of the LYWC group, 13,624 genes have been modified by m6A, and in the samples of the SIMC group, 24,522 genes have been modified by m6A. We found that among the differentially expressed genes, 620 genes were significantly expressed. Based on this, we screened 316 genes with significant differential expression and m6A methylation modification. The result is shown in Figure 5. Since this experiment mainly explored the regulation of muscle growth and development, we screened four candidate genes related to muscle cell growth and development, as shown in Table 6. The m6A regulation of these genes was upregulated, while gene regulation was downregulated. At the same time, the difference between the two sets of samples were significant.

**TABLE 6:** M6A -modified candidate genes related to muscle cell growth and development.

Gene name	Gene ID	M6A regulation	Gene regulation	FPKM.LYWC_input			FPKM.SIM_input		
				LYWC1	LYWC2	LYWC3	SIM1	SIM2	SIM3
TNNT1	282095	up	Down	6852.32	4722.65	7863.95	14067.95	12547.06	20219.39
MYOM2	524077	up	Down	603.17	446.51	330.98	990.94	1472.69	395.06
XIRP1	509670	up	Down	235.30	353.27	241.41	1260.87	1205.70	609.67
MYH6	100296004	up	Down	156.23	115.56	364.75	508.30	365.54	564.40

### GO Analysis and KEGG Pathway Analysis of Differentially Expressed m6A Methylation Genes

**FIGURE 6:**
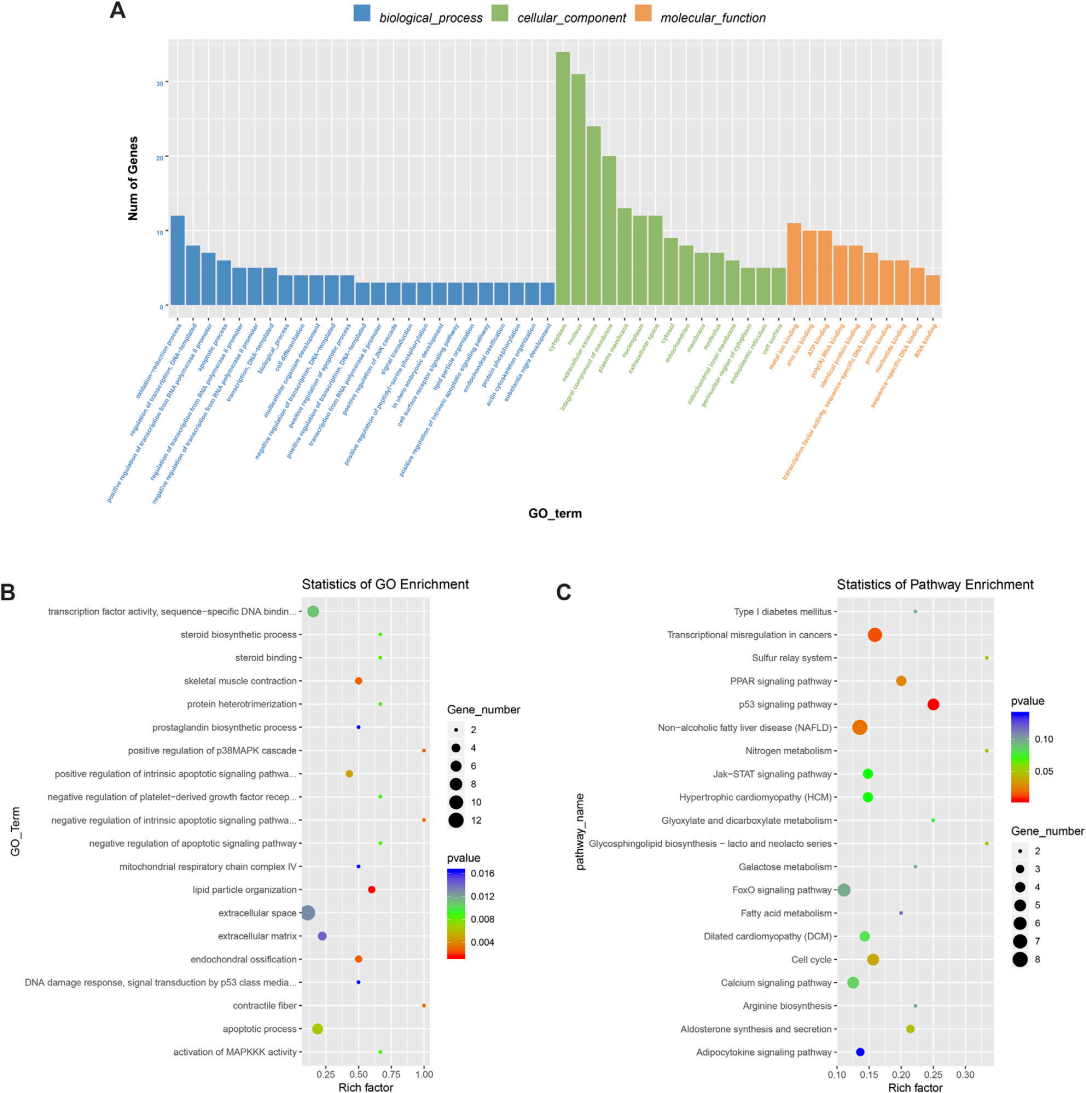
M6A differential peak gene ontology enrichment analysis and KEGG pathway analysis. **(A)** Main enrichment 3 and meaningful GO entries of m6Apeak. **(B)** The first 20 items have significantly enrichment GO terms. **(C)** The first 20 enriched pathways of the m6A peak.

To deeply study the significance of m6A modification in physiological and biochemical processes, we conducted GO (http://www.geneontology.org/) and KEGG (http://www.kegg.jp/) analyses of the different peaks of m6A. The peaks selected were enriched with 898 GO items and 162 pathways. Figure 6A shows the top 25 items in biological processes, the top 15 items in cell components, and the top 10 items in molecular functions. GO analysis (Figure 6B) showed that differentially methylated genes significantly enriched fibers in fat granule tissue, skeletal muscle contraction, and muscle contraction. KEGG pathway analysis (Figure 6C) showed that differentially methylated genes were related to the p53 signaling pathway and PPAR pathway. At the same time, they are also involved in biological processes such as galactose metabolism, fatty acid metabolism, adipocytokine pathway, nitrogen metabolism, arginine synthesis, etc.

### Verification of Differentially Expressed Genes

**FIGURE 7:**
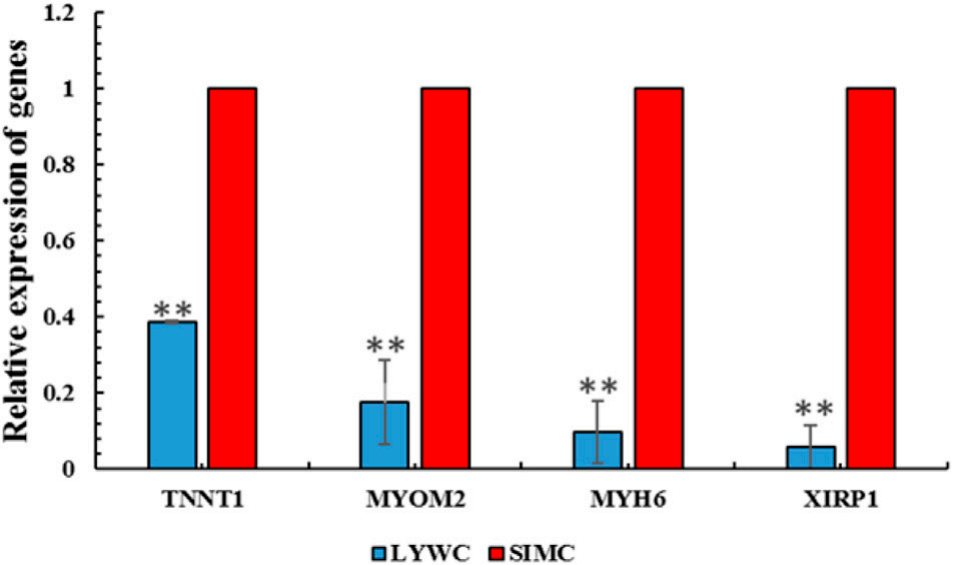
QPCR results of four different m6A-modified genes in LYWC and SIMC.

To study the function of gene m6A modification and determine the key genes that regulate muscle growth and development in beef cattle muscle cells, We used qRT-PCR for experimental verification. RNA-seq results showed that among the screened differential genes, the expression of Liaoyu white cattle group was lower than that of Simmental cattle group. The qRT-PCR results also confirmed that the m6A-modified gene is indeed present in the Liaoyu white cattle muscle. The trends of these genes are consistent with the RNA-seq results (Figure 7).

## Discussion

The modification of m6A is involved in many physiological processes, such as: Mediates mRNA output and synthesis, affects cell maturation, lipogenesis, maintains embryonic development stability, affects cell circadian rhythm, regulates stem cell differentiation, maintains Tregs stability, participates in inflammatory response, apoptosis, muscle production, cell Physiological and biochemical processes such as division. At the same time, the modification of RNA methylation is a dynamic change (Jia et al., 2011)^,^ (Zheng et al., 2013)^-^ (Feng et al., 2018).m6A RNA modification is dynamically regulated by methyltransferases (writers) and demethylases (erasers). Since m6A bases cannot be directly detected by sequencing, the dissection of the m6A landscape is hindered; they do not change the base pairing properties and cannot be distinguished from conventional bases by reverse transcription (Brocard et al., 2017)^.^ Recently, new methods based on m6A immunoprecipitation or modified selective RNA chemistry methods to isolate modified RNA fragments coupled with high-throughput sequencing, namely, m6A-seq and MeRIP-seq, have identified thousands of hundred-nucleotide fragments containing modifications in the transcriptomes of mammalian cells (Dominissini et al., 2012; Meyer et al., 2012). Modification of m6A has been successively discovered in many animals, plants, bacteria and other microorganisms.

Based on numerous research reports, it is found that the majority of cows mammary gland and lymphocytes undergo m6A modification phenomenon (Burtseva et al., 1979; Horowitz et al., 1984; Wu et al., 2021). However, there are still few reports on beef cattle. We speculate that m6A is involved in the muscle growth and development process of beef cattle. Our data show that there are a large number of methylation modifications during muscle growth and development. It may have an important effect on the types of muscle fibers, the maturation of muscle cells, and the changes in muscle structure.

Through laser-induced microdamage of zebrafish muscles combined with cell repair, it was found that the XIRP1 gene is abundant in skeletal muscle and involved in cell repair, cells and new myofibrils, and the repair of damage does not involve cell proliferation (Otten et al., 2012). Troponin T (TNNT1) exists as a group of homologous proteins in the striated muscle of vertebrates and invertebrates. Mutations in the TNNT1 gene can cause rod-shaped myopathy. From animal model experiments, it was found that lack of TNNT1 reduced the content of slow fibers, accompanied by type II fiber hypertrophic growth and increased muscle fatigue (Feng et al., 2009; Wei et al., 2014). Myosin 2 (MYOM2) is the main component of the myofibril M-band of the sarcomere and the central gene in the interaction of sarcomere genes (Auxerre-Plantie et al., 2020). Research by Auxerre-Plantié found that loss of function and moderate knockdown of this gene can lead to myocardial expansion, and severe knockdown can lead to increased sarcomeric myosin (Auxerre-Plantie et al., 2020). Research by Andrei found that in hypothyroid rats, MYOM2 expression increased 3.4 times. Through small-molecule interference of RNA with MYOM2, it was found that the contraction speed of cardiomyocytes were severely reduced (Rozanski et al., 2013). Myosin heavy chain 6 (MYH6) is widely found mainly in the heart and smooth muscle. This gene is mainly expressed in type I fibers. The presence or absence of MYH6 and its family gene MYH7 determines the slow or fast-twitch phenotype of skeletal muscle (Stuart et al., 2016).

Based on GO enrichment and KEGG pathway analysis, we speculate that m6A modification in genes has potentially important functions and may play a vital role in certain pathways are involved in cell growth and development. Peroxisome proliferator-activated receptors (PPARs) are nuclear hormone receptors activated by fatty acids and their derivatives. They are ligand-activated receptors in the nuclear hormone receptor family. Three subtypes have been found in different species, which control many intracellular metabolic processes. The subtypes include PPARα (also known as NR1C1). PPARα participates in the liver and skeletal muscle through regulation and expression of lipid metabolism genes. PPARβ/δ participates in lipid oxidation and cell proliferation. PPARγ promotes the differentiation of adipocytes to enhance blood glucose uptake. PPAR transcriptional activity can be regulated by nongene crosstalk with phosphatases and kinases, including ERK1/2, p38-MAPK, PKA, PKC, AMPK and GSK3. At the same time, nuclear receptor coactivator (coactivator) and PPAR-RXR act synergistically and complement and stabilize the active transcription complex, which can regulate lipid metabolism and fat formation, maintain metabolic homeostasis and the expression of inflammation genes, and induce anticancer effects in a variety of human tumors.

The m6A regulation level of the differentially expressed genes screened in this study was negatively correlated with the transcription level. The RNA-seq results showed that the differential gene expression in LYWC was lower than that in SIM. The results of qRT-PCR showed that the differentially expressed genes for m6A methylation were all present in the muscle tissue of beef cattle. Therefore, this indicates that m6A modification not only participates in the process of muscle growth and development but may also regulate mRNA degradation.

Skeletal muscle development is a complex biological process. The regulatory role of myogenic regulatory factors and the study of apparent modifications, including DNA methylation and histone modification, in the regulation of skeletal muscle development have given us a preliminary understanding of the regulatory network of skeletal muscle development. Based on the involvement of m6A in the regulation of mouse brain development, fat formation, and other tissue development processes, we speculate that m6A is also involved in the regulation of skeletal muscle development (Zhao et al., 2014; Ma et al., 2018; Wang et al., 2018). Studies have shown that the regulation of METTL3 gene expression and regulation of m6A levels in myoblasts affect the differentiation process of myocytes and the expression of key regulatory genes (Kudou et al., 2017; Chen et al., 2019). This shows that m6A is involved in the regulation of muscle cell differentiation. During the development of animal embryos and the growth and development of brain tissue after birth, neural stem cells are required for differentiation and self-renewal. Studies have shown that knocking out METTL14 in mouse embryos will interrupt the cycle of radial glial cells in the nerves, which will eventually lead to a decrease in the thickness of the cerebral cortex and even death after birth. Overexpression and specific knockout of the FTO and METTL3 genes in pig adipocytes revealed that FTO expression levels are negatively correlated with m6A levels and positively correlated with adipogenesis, while METTL3 expression levels are positively correlated with m6A levels and negatively correlated with adipogenesis (Wang et al., 2015b). Mice lacking FTO function experience increased energy expenditure, growth retardation, lean body size after birth and deformity (Boissel et al., 2009). Through transcriptome sequencing of the muscle tissues of three different breeds of wild boar, Landrace pig and Rongchang pig, a complete transcriptome map of m6A was drawn. It was found that m6A is widely distributed in muscle tissue, and m6A is mainly enriched in related gene stop codons, 3′UTRs, and protein coding-regions. In addition, data show that there is a clear m6A peak around the stop codon of the cAMP response element-binding protein CREB and zinc finger protein ZNF-related genes, indicating that m6A is enriched here. CREB was first discovered as a transcriptional regulator of cell metabolism that regulates the cAMP response. It is an important gene regulating somatostatin. ZNF has also been considered one of the most important eukaryotic transcription factors and plays an important role in gene regulation.

In conclusion, this study analyzed m6A methylation modification in the muscle tissue of Liaoyu white cattle and Simmental cattle. Based on our experimental results, we speculate that m6A modification plays an important role in muscle growth and development. This study shows that TNNT1, XIRP1, MYOM2, and MYH6 are likely to play a key role in muscle growth and muscle differentiation. In addition, the data obtained through high-throughput sequencing provide a theoretical basis for further exploring the function of m6A modification on muscle growth and development. At the same time, the regulatory mechanism of m6A modification in muscle still needs to be studied in depth in the future.

## Data Availability

The datasets presented in this study can be found in online repositories. The names of the repository/repositories and accession number(s) can be found below: NCBI [accession: PRJNA778440].
